# Analysis of bone healing with a novel bone wax substitute compared with bone wax in a porcine bone defect model

**DOI:** 10.4155/fsoa-2018-0004

**Published:** 2018-07-26

**Authors:** Tristan Tham, Keith Roberts, John Shanahan, John Burban, Peter Costantino

**Affiliations:** 1 New York Head & Neck Institute, Lenox Hill Hospital, New York, NY, 10075, USA; 2 Hemostasis LLC, St Paul, MN, 55110, USA

**Keywords:** biocompatibility, biomaterials, bone wax hydroxyapatite, hemostasis, osteoconduction, porcine model

## Abstract

**Aim:**

This pilot study describes a novel composite of hydroxyapatite and biodegradable polylactic acid with wax-like handling properties (BoneSeal^®^). The goal was to compare quantitative measures of bone healing between BoneSeal versus Bone wax.

**Materials & methods:**

BoneSeal and Bone wax were introduced into separate defects of a single porcine specimen. After 6 weeks, the defect sites were harvested for analysis.

**Results:**

Both groups had similar hemostatic action. The amount of new bone was significantly greater at 6 weeks in the BoneSeal group (38.05%) versus the Bone wax group (11.88%), p = 0.028.

**Conclusion:**

In this pilot study, BoneSeal had higher amounts of new bone formation compared with Bone wax.

## Lay abstract

This small pilot study compares two materials, Bone wax and BoneSeal, which could be used in bone surgery. In bone surgery, there is a need for the materials to stop bleeding as well as have good bone healing capabilities. We investigated the bone healing characteristics of two different materials. We implanted these materials into the arm and leg bones of a single pig. After a period of 6 weeks, we investigated the sites of implantation. We found that implantation sites of BoneSeal had higher amounts of new bone formation.

Bone wax, first described by Sir Victor Horsley as an antiseptic in 1892, is now commonly used in surgery as a hemostatic device [1,2]. In cardiothoracic surgery, the main indication for Bone wax is extensive bleeding from the sternal bone marrow. During sternotomy procedures, intraoperative exposure of the cut surfaces of the sternum, together with concurrent anticoagulant therapies increases the patients’ risk of bleeding into the sternal wound. Surgeons try to minimize such bleeding as the eventual intrasternal hematoma collection could serve as a nidus for bacterial infection, an uncommon but serious sequelae of cardiothoracic surgery [3]. In order to prevent extensive hematoma formation, surgeons apply topical hemostatic agents, such as Bone wax, to the cut sternal marrow [4]. Surgical Bone wax consists of the naturally occurring sterilized *Cera Alba* (honeybee wax), mixed with paraffin, a softening agent.

Since Bone wax is comprised of paraffin and esterified fatty acids, it is highly hydrophobic. This enables Bone wax to serve as a physical barrier against blood, occluding the bleeding channels and achieving hemostasis by a tamponade and blood stasis effect. However, this hydrophobic property in conjunction with limited enzymatic degradation of waxes in the human body prevents appreciable rates of absorption and/or excretion postsurgical application. It is well documented that Bone wax impairs optimal bone formation and healing of sternotomies [3,5], which could be due to, in part, physical inhibition of osteoblast and osteocyte migration to the site of bony injury. Furthermore, the nature of Bone wax which makes it highly resistant to degradation has also been linked with infection [6,7], although large randomized studies have found the infection link to be inconclusive [8]. Since intraoperative bone bleeding can be heavy, physicians must weigh the benefits of bone hemostasis using Bone wax versus the risk of decreased bone healing and other complications such as infection.

It is important that Bone wax-like substances have biocompatible, osteoconductive, hemostatic, as well as good handling properties. Several alternative materials have been reported in the literature such as PEG/collagen [9], polyorthoester [10], fibrin-collagen [11], chitin-based material [12] and gelfoam [13]. However, none of these alternative materials have yet seen widespread adoption, suggesting that a material which meets the effective hemostatic qualities of Bone wax together with good osseous integration and affordability has not been met.

Herein we describe BoneSeal^®^ (Hemostasis LLC, MN, USA), a novel composite of hydroxyapatite (HA) and biodegradable polylactic acid (PLA) with wax-like handling properties. HA (*Ca10(PO4)6(OH)2*) is a biomaterial similar to the mineral component of natural bone, and exhibits good osteoconductivity [14]. Similar to Bone wax, the BoneSeal creates a physical barrier which is its primary mechanism of hemostasis. Unlike Bone wax, we hypothesized that the BoneSeal would, due to its HA content, have an enhanced bone healing profile in addition to hemostatic qualities. The goal of this pilot experiment was to compare the widely used cardiothoracic surgery hemostatic agent, Bone wax, versus BoneSeal. We investigated qualitative and quantitative measures of bone healing between the two materials.

## Materials & methods

Details of animal husbandry, diet, care, monitoring, health and well-being as well as measures to alleviate suffering were all performed in accordance with International Organization for Standardization document ISO 10993–11:2006: Biological evaluation of medical devices. This experiment was conducted in a Sichuan Province Experimental Animal Management Committee Accredited Facility in PR China. Ethics approval for animal experimentation was approved by the animal ethics board of the Sichuan Province Experimental Animal Management Committee of China (Approval number 2015–05128NG).

A porcine bone defect model was used for this study. One female young adult Landrace pig weighing 52.8 kg was used for this study, with an acclimation period of 7 days. Two groups of holes were drilled into the femur and humerus and filled with either BoneSeal or Bone wax.

Bone Wax (Bone Wax, Ethicon, NJ, USA) is a mixture of beeswax, paraffin and isopropyl palmitate, a wax-softening agent. It is currently widely used as a method of controlling bleeding from bone surfaces and achieves local hemostasis of bone by acting as a mechanical barrier. It does not act biochemically and is nonabsorbable. Further details of the properties of Bone Wax have been published by others [15]. As described above, BoneSeal (Hemostasis LLC) is a novel composite of HA and biodegradable PLA. It is primarily composed of 85–90% of a proprietary formulation of synthetic PLA polymer. The remaining 10–15% is composed of HA (*Ca10(PO4)6(OH)2*) particles. After a period of 6 weeks, qualitative (clinical and histology) and quantitative (histomorphometry) analyses were performed on the drilled sites.

### Surgical procedure

The animal was prepared for operation under general anesthesia. Intraperitoneal pentobarbital sodium was administered and the field of operation was then sterilized and selected at the right humerus and contralateral left femur. Tissue dissection was performed, exposing the underlying periosteum at these respective sites. Four test sites were chosen for the bone defect, two holes located in the right diaphyseal humerus and two holes located in the left diaphyseal femur. All test sites were drilled to have standardized intraosseous defects with a circumference of 3 mm and equal depth. Choice of defect diameter was based on previous experimental models as well as common sternum screw sizes in cardiothoracic surgery [16–18]. Care was taken to drill the cortical bone down to a similar depth in all the drill sites. For each hole, approximately 35–50 mm^3^ of material was used, enough to fill each drilled hole. The material was manually applied by hand, in sterile fashion, to fill each drilled hole with material until it was flush with the surface of the external cortex. The dimensions of the drilled hole were based on previous studies in various other experimental models. Furthermore, cortical screws in cardiothoracic surgery often use 3 mm sized holes which was another rationale for the selection of the defect size. One defect on each bone (humerus or femur) was filled with BoneSeal and the other defect was filled with Bone wax W31G (Ethicon, NJ, USA). The surgical sites were closed in multiple layers.

The animal was given a healing period of 6 weeks before it was sacrificed. During the healing period, the animal displayed no signs of local infection and surgical site incisions were well healed. The animal was fully ambulant during the entire period and was effectively load bearing on all four limbs. After the healing period, the animal was sacrificed with an overdose of pentobarbital sodium. The right humerus and left femur diaphysis were cut to harvest the test sites as discrete blocks. On harvesting of the test sites, no signs of gross inflammation or necrosis were observed in any of the sites.

### Histology preparation

The harvested blocks of tissue containing the test sites were fixed with a 10% neutral buffered solution of formalin for a period of 7 days. The blocks were then decalcified by a solution of mixed acid decalcification agent for 6 weeks. Next, the sections were dehydrated with ethanol gradient and prepared with paraffin. Hematoxylin and eosin stain was applied to the sections for histological observation at ×40, ×100 and ×400 magnifications. Two sections were prepared from each of the 4 drilled sites, for a total of eight sections for histological/histomorphometric analysis (four sections for the BoneSeal defect and four sections for the Bone wax defect).

### Histomorphometric evaluation

Two sections per drilled site were used for histomorphometric analysis, for a total of four sections per test material. Analysis was done at the ×40 power magnification level, as this would give a balance between tissue resolution and reduction in section variability, which would be greatly biased at higher power magnification. Histomorphometric data were obtained by analyzing the magnified cross sections of the defect sites using National Institute of Health program software, ImageJ 1.48 v. Areas of interest were measured in terms of pixel counts. Parameters delineated in the histomorphometric evaluation were percent bone area, percent soft tissue area, and percent test material, all of which combined would add up to approximately 100% of the defect size.

### Statistical analysis

Histomorphometric data were analyzed by comparing the mean between the two test materials using Student's *t*-test. The null hypothesis is that the amount of new bone formation is the same between the BoneSeal group versus the Control group. Results were considered significant with a p-value of less than 0.05.

## Results

### Clinical evaluation

Upon drilling defects into the femoral and humeral diaphysis, bone bleeds were observed in all the bone defects. There was effective hemostasis in both materials. The animal remained healthy for the duration of the study with no postoperative or surgical site complications. The rest of the 6 week healing period was uneventful.

### Histology

A selection of images which are representative of the qualitative findings are included below.

#### ×40 power magnification

**Figure 1.**
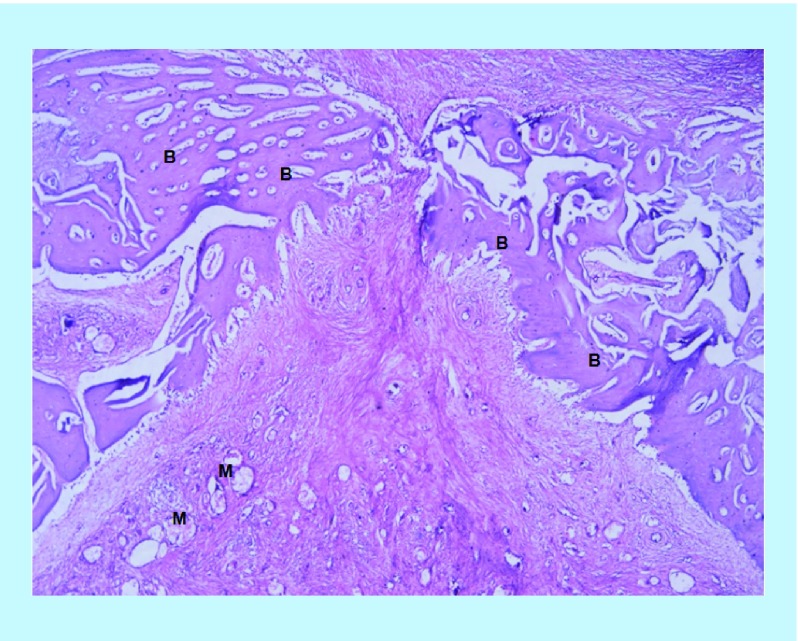
**Hematoxylin- and eosin-stained view of the bony defect containing BoneSeal^®^ at ×40 power magnification.** Formation of new bone can be seen on the lateral and inferior borders of the image. Haversian/Volkmann-like vessels are present in the central portion of the image. Fibrous stroma is also present in the middle of the section at the interface of the newly formed bone. Some undegraded material is present. B: New bone; M: Undegraded material.

**Figure 2.**
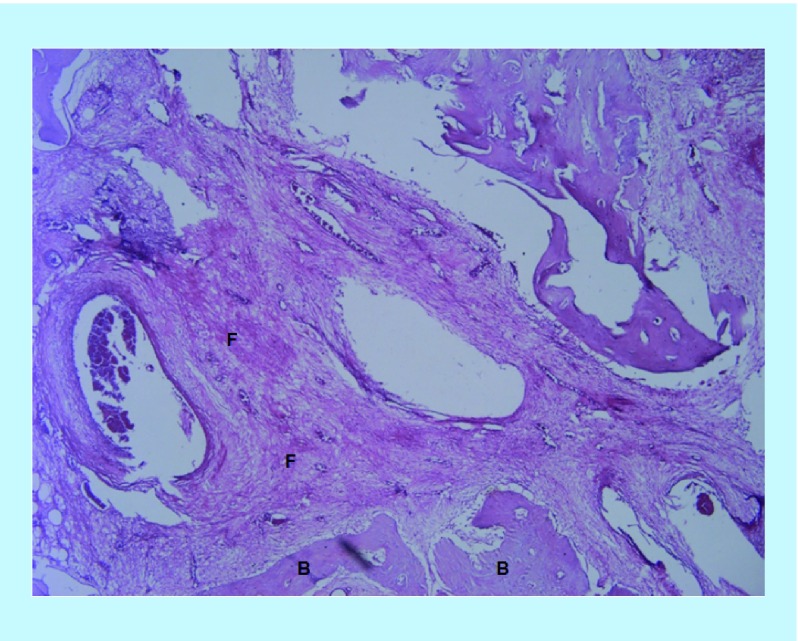
**Hematoxylin and eosin section containing Bone wax at ×40 power magnification.** This section is dominated by fibrous stroma in the middle of the image, and a small amount of new bone formation at the peripheries. B: New bone; F: Fibrous stroma.

In the BoneSeal sites, new bone formation was observed, in addition to the soft tissue stroma. Furthermore, new vessel formation was observed in some of the sections (Figure 1). In the Bone wax sites, fibrous soft tissue stroma was the predominating component. Some new bone can also be seen, and there is also the presence of undegraded test material. The amount of new bone appears visually less than in the BoneSeal sites (Figure 2).

#### *X100 power magnification*

**Figure 3.**
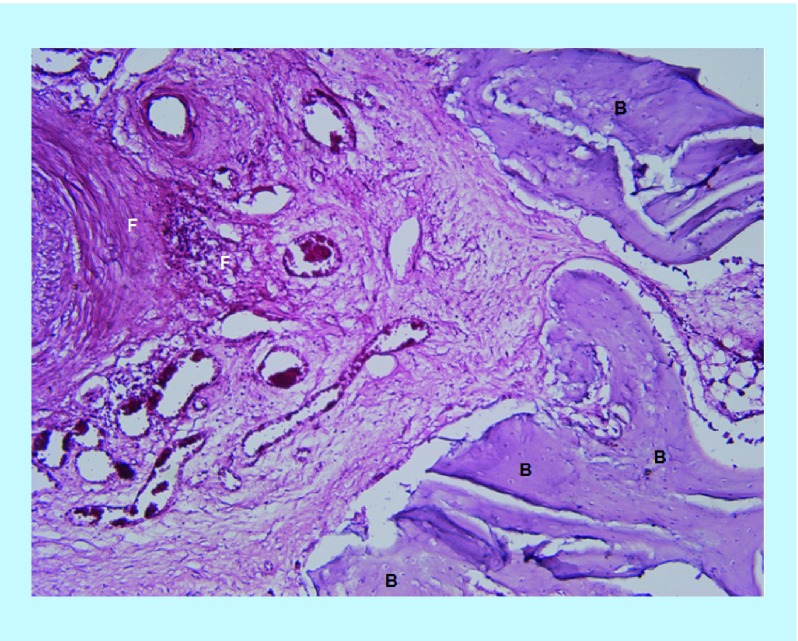
**Section of BoneSeal site at ×100 power magnification.** New bone can be seen on the right margins, with the left margin composed of fibrous stroma. B: New bone; F: Fibrous stroma.

**Figure 4.**
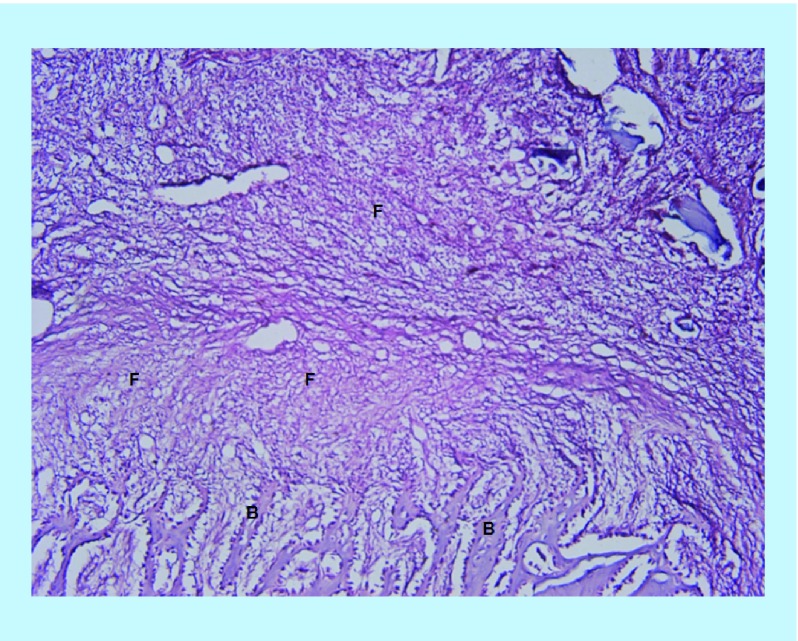
**Section of the Bone wax site at ×100 power magnification.** Immature bony trabeculae are seen at the inferior margins. The rest of the image is mainly composed of fibrous stroma. B: bony trabeculae; F: Fibrous stroma

In the BoneSeal sites, new bone formation can be seen which make up a large portions of the images (Figure 3). Bone wax sites show a large amount of fibrous tissue and some new bone is also present, but as immature bony trabeculae (Figure 4).

#### *×400 power magnification*

**Figure 5.**
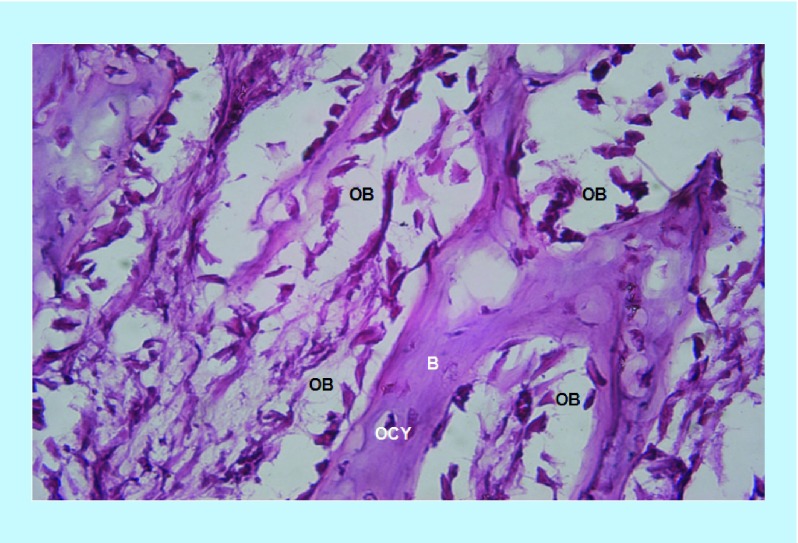
**BoneSeal site at ×400 power magnification.** Osteoblasts are seen lining the newly formed bony trabeculae, with osteocytes also present in the bony matrix. B: bony trabeculae; OB: Osteoblasts; OCY: Osteocytes.

**Figure 6.**
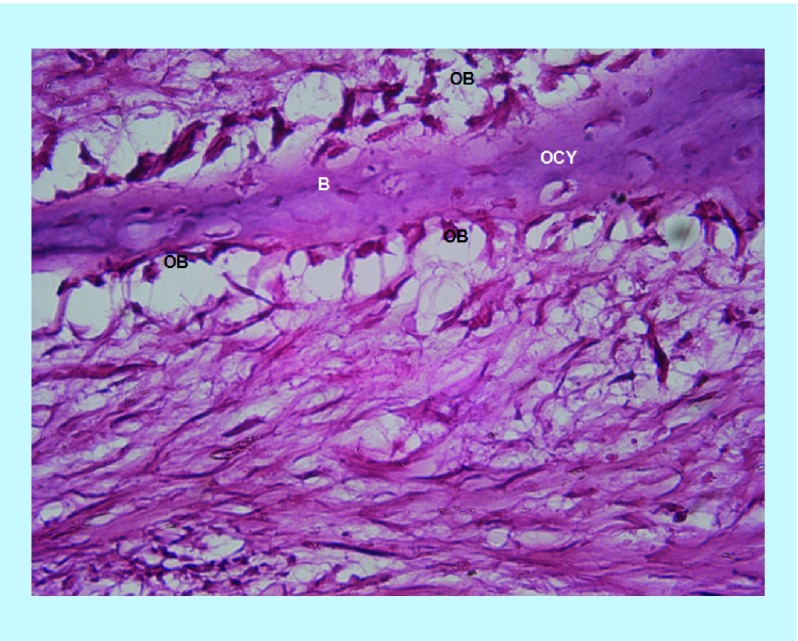
**Bone wax site at ×400 power magnification.** Similar to Figure 5, osteoblasts are seen lining the newly formed bony trabeculae, with osteocytes also present in the bony matrix. The bottom portion of the image consists of soft tissue. B: bony trabeculae; OB: Osteoblasts; OCY: Osteocytes.

BoneSeal sites shows active osteoblast activity lining the bony trabeculae. Osteocytes are also present in the bony matrix. No osteoclasts are seen (Figure 5). Similarly, the Bone wax site (Figure 6) shows osteoblast activity lining the bony trabeculae, with no osteoclasts. The variation in sections at the ×400 magnification is inherently greater.

### Histomorphometry

**Figure 7.**
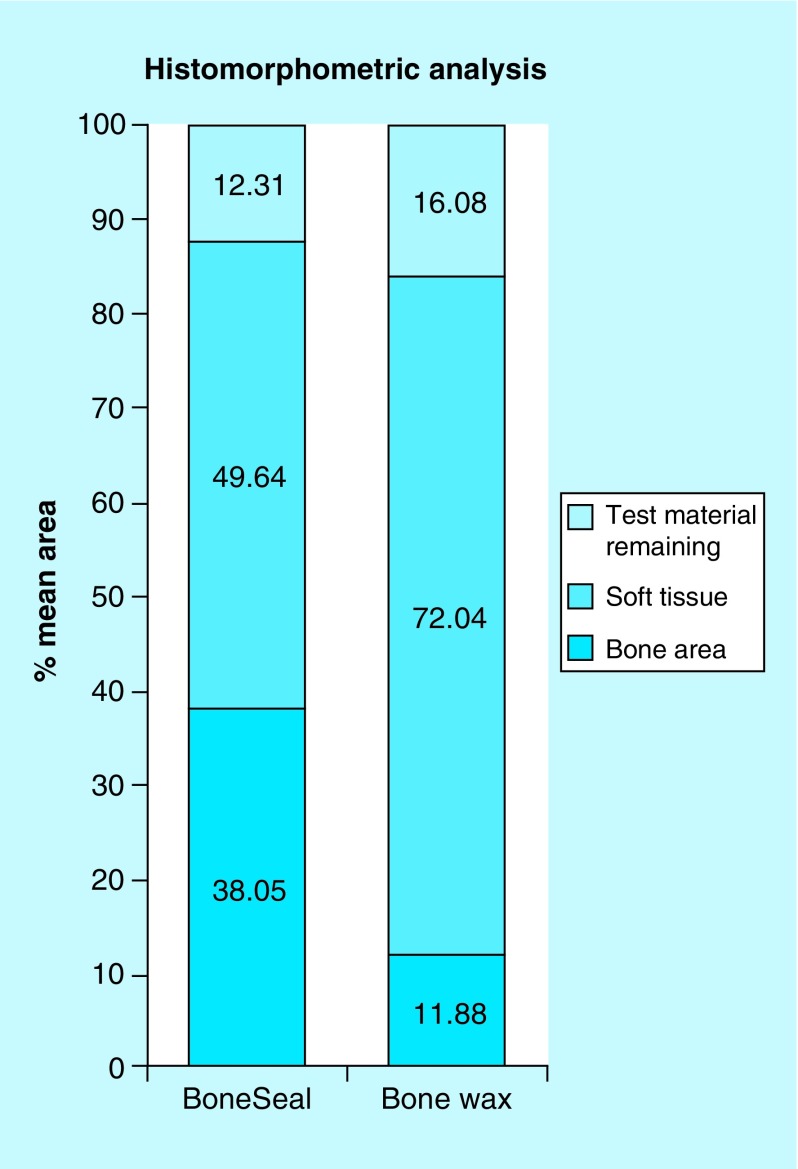
**Histomorphometric analysis of BoneSeal^®^ versus Bone wax.** Histomorphometric analysis of the sections at ×40 power magnification. Parameters measured were mean volume fraction of test material remaining, soft tissue and bone. All area fractions add up to make 100% of the total volume. The difference in areas of bone (p = 0.028) between the two groups are statistically significant. The difference in areas of soft tissue (p = 0.089) and test material remaining (p = 0.421) were not statistically significant.

Histomorphometric analysis of the sections at ×40 power magnification was performed. Parameters measured were mean volume fraction of test material remaining, soft tissue area and bone area. The mean area fractions of test material, soft tissue and bone in the BoneSeal composite were 12.31, 49.64 and 38.05%, respectively. In the Bone wax group, the mean area fractions of test material, soft tissue and bone were 16.08, 72.04 and 11.88%, respectively (Figure 7). The difference in bone area fraction between the BoneSeal group (38.05%) and the Bone wax group (11.88%) was significant (p = 0.028) (Table 1). The soft tissue area fraction in the BoneSeal group (49.64%) was also less than the Bone wax group (72.04%); however, this result was not statistically significant (p = 0.089). Similarly, the amount of test material remaining in the BoneSeal group (12.31%) was less than the Bone wax group (16.08%) and was also not statistically significant (p = 0.421).

**Table 1.** **Bone area seen in histomorphometric analysis in BoneSeal^®^ versus Bone wax.**

**Sample**	**BoneSeal bone area (%)**	**Sample**	**Bone wax bone area (%)**
Sample 1	52.49	Sample 1	9.65

Sample 2	12.42	Sample 2	15.96

Sample 3	44.31	Sample 3	6.45

Sample 4	42.98	Sample 4	15.44

Mean	38.05^†^	Mean	11.88

Standard deviation	17.6	Standard deviation	4.61

Histomorphometric analysis of 4 samples of bone defects for BoneSeal and Bone wax. The mean amount of bone observed in histomorphometric analysis was significantly greater in BoneSeal compared with bone wax.

^†^p < 0.05

## Discussion

Histomorphometric data revealed that the amount of new bone was significantly greater at 6 weeks in the BoneSeal group (38.05%) versus the Bone wax group (11.88%), p = 0.028. These data are consistent with the published literature in that Bone wax is a potent inhibitor of bone healing [4,19]. Histological examinations in animal models as well as human autopsies demonstrate that the application of Bone wax not only prevents bone healing, but also promotes granuloma formation, chronic inflammation and fibrotic scar tissue [3,5,20]. Furthermore, Bone wax is resistant to degradation and remains in the implanted sites indefinitely [5,20]. In a case series of 18 postmortem examinations, histologically verified Bone wax granulomas were found, in one case as long as 10 years after implantation [5]. Because of these potential complications, good surgical practices minimize the amount of Bone wax used, whenever needed. Its use is avoided altogether when fusion of bone is critical for postoperative function, for example, in most orthopedic surgery procedures.

Bone wax hemostatic activity is purely mechanical. It physically occludes the bleeding haversian canals in cortical and medullary bone and activates coagulation via the stasis component of Virchow's Triad. The BoneSeal composite used in this experiment has similar handling characteristics to Bone wax, and was designed to promote hemostasis via a similar tamponade-like mechanism. Despite that no objective measures of hemostasis were designed as part of this study, both materials are subjectively reported to have equal hemostatic efficacy.

Our results showed that the amount of fibrous tissue remaining in the BoneSeal group (49.64%) was less than in the Bone wax group (72.04%), though this was not statistically significant (p = 0.089). Furthermore, the amount of test material remaining for the BoneSeal group (12.31%), though less than Bone wax (16.08%), was also not significant (p = 0.421). One possible explanation for these findings could be that the timeframe of 6 weeks was too short for all the HA particles to take part in resorption and osteointegration. As described earlier, it would be reasonable to assume that the Bone wax particles would be resistant to degradation and resorption. However, HA particles have been shown to have complete osseous integration over a period of time, especially when blended with PLA oligomer [21].

Subjectively, both BoneSeal and Bone wax had effective hemostatic action on the bleeding bone. However, in this experiment, we noted that BoneSeal has the added advantage of having a higher bone healing capacity compared with Bone wax. One other hemostatic Bone wax substitute that does not inhibit healing is water soluble Bone wax (WSW). WSW was first reported as ‘pluronic-based’ wax by Wang *et al*. in 2001, and similar to this study, demonstrated superior bone healing to Bone wax [22]. A subsequent study by Vestergaard *et al*. of a randomized trial in humans comparing WSW versus Bone wax found no differences in infection rates, but radiologic bone measurements indicated lower levels of bone healing in Bone wax. Additionally, the study surgeons commented the WSW had some drawbacks, specifically the need to reapply due to dissolution of the WSW and the need to heat the WSW product before application to make it more pliable to smear on trabecular bone surfaces [23].

Whether or not the lower levels of radiologic bone healing in Bone wax compared with WSW translate into real clinical effects such as sternal bone strength is a matter for debate, as demonstrated in long-term animal trials. A study comparing WSW and Bone wax in porcine sternotomies after a period of 6 months showed that although Bone wax had poorer histological and radiological outcomes, bone mechanical properties were similar. Sternal wounds closed with Bone wax were found to be weaker compared with a negative control, but no difference in sternal strength was observed between Bone wax and WSW [24].

Similar to WSW, BoneSeal has several advantages over Bone wax and other reported Bone wax substitutes. BoneSeal used in this study has demonstrated a higher amount of bone growth, is easily sterilizable, is biocompatible and would theoretically fully integrate in bony architecture over a longer period of time [25]. Furthermore, since it has no biological components, the material would be immunogenic and allergen free. Physical handling characteristics which are similar to Bone wax would make BoneSeal easy to use for surgeons familiar with working with traditional Bone wax.

Our results need to be interpreted with caution due to several drawbacks. First, since this was a pilot study, our goal was to test the materials in a single animal. The single animal could predispose our results to a degree of bias. Second, we did not examine the quantitative hemostatic characteristics of both materials. Both materials were reported by the surgeon to be subjectively similar in terms of hemostatic ability, but this is limited due to observer subjectivity. Third, it is possible that the acid decalcification process used in the histological preparation would have degraded some HA that would otherwise be present at the end of the healing period. The holes displayed in the histological sections might be accounted for by calculating the percentage of excess material, but this is prone to bias. Fourth, as this pilot study was designed to investigate the bone healing properties of the materials, detailed physical property testing (porosity and morphology) of the materials was outside the scope of this study.

In conclusion, this pilot study has demonstrated that at 6 weeks, porcine bone defects will have higher amounts of new bone if filled with BoneSeal than with Bone wax. BoneSeal test sites also demonstrated less soft tissue and test material remaining than the Bone Wax, though the results for these parameters did not meet the threshold for statistical significance. This might be because the time frame for eventual HA osseous integration lasts many months to years. Our findings need to be interpreted with caution due to the limitations described above. Subjectively, both BoneSeal and Bone wax have effective hemostatic properties. It is important that bone hemostat substances or its substitutes have biocompatible, osteoconductive, as well as hemostatic properties.

## Future perspective

Further studies are needed to ascertain the efficacy of BoneSeal in the human sternotomy and/or cranial/spine surgery models, particularly comparing it against Bone wax and other common hemostatic agents. Parameters for future studies should focus on both hemostatic and bone healing characteristics. Other useful avenues for investigation would be longer term studies to ascertain the rate of HA integration in bone after application as a hemostat.

**Executive summary**

- Bone wax is a commonly used hemostatic agent in bone surgeries. However, it is resistant to biodegradation and resorption and thus imparts poor bone healing characteristics.
- In this study, we compare the bone healing characteristics of Bone wax versus a novel wax-like composite of hydroxyapatite and polylactic acid, BoneSeal.
- Our results show higher new bone formation in the BoneSeal group.
- Results need to be interpreted with caution due to the pilot nature of the study.
